# Sharpening coarse-to-fine stereo vision by perceptual learning: asymmetric transfer across the spatial frequency spectrum

**DOI:** 10.1098/rsos.150523

**Published:** 2016-01-20

**Authors:** Roger W. Li, Truyet T. Tran, Ashley P. Craven, Tsz-Wing Leung, Sandy W. Chat, Dennis M. Levi

**Affiliations:** 1 School of Optometry, University of California, Berkeley, CA 94720, USA; 2 Helen Wills Neuroscience Institute, University of California, Berkeley, CA 94720, USA; 3 School of Optometry, The Hong Kong Polytechnic University, Kowloon, Hong Kong

**Keywords:** visual plasticity, stereopsis, vision enhancement, specificity, generalization

## Abstract

Neurons in the early visual cortex are finely tuned to different low-level visual features, forming a multi-channel system analysing the visual image formed on the retina in a parallel manner. However, little is known about the potential ‘cross-talk’ among these channels. Here, we systematically investigated whether stereoacuity, over a large range of target spatial frequencies, can be enhanced by perceptual learning. Using narrow-band visual stimuli, we found that practice with coarse (low spatial frequency) targets substantially improves performance, and that the improvement spreads from coarse to fine (high spatial frequency) three-dimensional perception, generalizing broadly across untrained spatial frequencies and orientations. Notably, we observed an asymmetric transfer of learning across the spatial frequency spectrum. The bandwidth of transfer was broader when training was at a high spatial frequency than at a low spatial frequency. Stereoacuity training is most beneficial when trained with fine targets. This broad transfer of stereoacuity learning contrasts with the highly specific learning reported for other basic visual functions. We also revealed strategies to boost learning outcomes ‘beyond-the-plateau’. Our investigations contribute to understanding the functional properties of the network subserving stereovision. The ability to generalize may provide a key principle for restoring impaired binocular vision in clinical situations.

## 1. Introduction

Stereopsis, resulting from the horizontal displacement of the two eyes (i.e. binocular disparity), adds a rich third dimension to the visual world, enabling the observer to discern the relative distances of objects with extraordinary accuracy. Under ideal conditions, stereo thresholds are much finer than the spatial grain of the retinal mosaic and are considered a hyperacuity [1], as small as a few arc seconds of visual angle. The neural computations involved in extracting the binocular disparity information from the two monocular images are largely based on cortical processing at multiple levels [2,3]. It appears that the neuronal mechanisms supporting stereoscopic vision are not hard-wired, but may be modifiable through experience [2,4,5]. There is earlier evidence demonstrating that perceptual learning can enhance stereoacuity in human adults [6], beyond the critical period for visual development [7,8]. Perceptual learning is the long-term improvement in visual function that results from repeated practice of a challenging visual task. Perceptual learning has attracted a great deal of attention in the past several decades, in large part because it has been shown to be highly specific to the orientation and spatial frequency of the trained visual stimuli [9–14]. There is still much debate about the mechanisms of perceptual learning for stereopsis and where the alternations may occur in the visual brain [15–21].

Neurons in the primary visual cortex are tuned to encode low-level visual information such as spatial frequency, orientation, retinotopic location, etc. [22]. Thus, a neuron in early visual cortex responds to a limited range of stimulus spatial frequencies, orientations and locations, forming the basis of a multi-channel system analysing the visual image formed on the retina. The properties of visual plasticity in the individual spatial frequency channels [23,24] are not yet clearly understood. We are particularly interested in whether there is ‘cross-talk’ between these putative channels. Here we ask whether perceptual learning of stereoscopic depth perception generalizes broadly across spatial frequency and orientation. This has important ramifications for clinical applications, because such vision training exercises would not be useful in everyday life if the learning effects do not transfer to other untrained stimuli. We are also interested in making the learning as efficient as possible. In order to determine the most efficient strategy, we compare the transfer of learning from high spatial frequency to low and vice versa. In this study, we systematically quantified the effects of perceptual learning using narrow-band visual stimuli, spanning a large spatial frequency range. Using a multi-stage training protocol, we characterized the magnitude and specificity of learning with respect to low-level, basic visual features, providing new insights into the mechanisms of neural plasticity and importantly, the strategies to optimize visual performance. Our investigations contribute to understanding the hierarchical architecture and functional properties of the network subserving stereovision.

## 2. Material and methods

### 2.1 Subject

A total of 31 healthy young adults, ages 20–40 years, participated in three groups. All had normal or corrected-to-normal visual acuity of 20/16^−2^ or better in each eye; the interocular acuity difference was two letters or less on a standard LogMAR letter chart (National Vision Research Institute of Australia, 1978). Inclusion criteria were spherical refractive error in the range of +2D to −5D and astigmatism in the range of 0–0.50D. None of the participants had anisometropia of greater than 1D spherical equivalent difference between the two eyes. They had neither strabismus nor amblyopia. All participants had normal stereoacuity of 40 arcsec or better (Randot^®^ stereotest, Stereo Optical Co., Inc., Chicago, IL, USA) and the heterophoria, if any, was within the normal range at distance and near as examined by alternate cover test. All testing and training was done with the observer wearing best optical correction.

### 2.2 Visual stimulus

**Figure 1.**
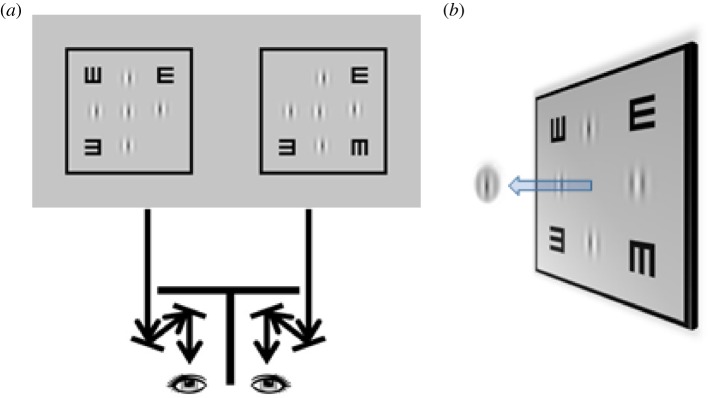
Stereo stimulus. (*a*) The stereogram consisted of two slightly different pictures—one to each eye. At the centre each square was a target Gabor patch surrounded by four reference Gabor patches. To eliminate any possible monocular cues, the vertical and horizontal coordinates of each Gabor patch and also the patch features, the carrier phase, were randomly jittered according to a uniform distribution. A custom-built mirror stereoscope was used to view the stereo pairs, so that the left eye would see the left square and the right eye would see the right one. Binocular disparity was generated by shifting the two target Gabor patches, one on each side, horizontally in opposite directions (uncrossed disparity, both shifted temporally; crossed disparity, both shifted nasally). (*b*) Binocular fusion of the two monocular images creates a cyclopean image. The visual task was to determine the stereoscopic depth of the target Gabor (in front/behind) relative to the four adjacent references. This schematic diagram illustrates crossed disparity—the target Gabor patch appeared in front of the reference patches.

The visual stimulus consisted of two horizontally separated black squares. At the centre of each square was a target Gabor patch surrounded by four reference Gabor patches with the same spatial frequency and orientation as the target (figure 1*a*). A custom-built 4-mirror stereoscope was used to present a half monitor screen to each eye (i.e. the left square for the left eye and vice versa). Binocular disparity was introduced by shifting the two target Gabor patches (one in each square) in opposite directions. The position and phase of each Gabor patch, both target and reference, were randomly jittered based on a uniform distribution (vertical and horizontal position range, ±20 screen pixels—more than an order of magnitude larger than the observers’ stereo thresholds; phase range, 0–360^°^) to minimize any possible monocular cues, for example, Vernier and bisection cues. The following abbreviations are adopted to describe the Gabor patch parameters: V1, V5, V10, V20 and H5, where the letter describes the carrier orientation (V, vertical; H, horizontal) and the number specifies the carrier spatial frequency expressed in cycles per degree.

### 2.3 Cyclopean view

Figure 1*b* illustrates the cyclopean percept of the visual targets in binocular viewing. The outer square served as a fusion lock to ensure proper alignment of the two eyes. The two letter Es at the top right and bottom left corners served as an accommodation lock for stimulating accommodation to bring the visual stimulus in focus. The E at the top left corner and the other E at the bottom right corner served as a binocular indicator ensuring the absence of monocular suppression during testing, and proper alignment (when perceived to be one above the other). Observers were given careful instructions for adjusting the haploscope mirrors in order to avoid stimulating excessive convergence, which could trigger convergence-accommodation and result in blurred vision. The visual task was to determine the stereoscopic depth of the target Gabor (in front or behind) relative to the four reference Gabor patches. Trial-by-trial audio feedback was provided for each response.

### 2.4 Stimulus scaling

The stimulus spatial scale was manipulated by changing the physical stimulus size on the screen and varying the viewing distance, 50 cm (V1) or 2 m (V5, V10, V20 and H5). As for V5 stimuli viewed at 2 m, the s.d. of the Gaussian envelope was 7 arcmin, Gabor centre-to-centre distance was 48 arcmin when positional jittering was off, the letter ‘E’ size was 25 arcmin and the square was 197.3 arcmin. The carrier spatial frequency and Gaussian envelope s.d. covaried, keeping a constant number of cycles/s.d. All visual stimuli were displayed on a 21-in Sony F520 flat monitor screen at 1800×1440 resolution and 90 Hz refresh rate. The mean luminance of the stimuli was 55 cd m^−2^ and the contrast of each Gabor patch was 99%. Light shielding was used to block stray light from the monitor screen. The inter-pixel distance was 20 arcsec at a viewing distance of 2 m (50 cm, 80 arcsec); sub-pixel accuracy was achieved by contrast manipulation.

### 2.5 Psychophysical methods

For each trial, the amount of binocular disparity was controlled by two interleaved adaptive staircases to track the stereo threshold: one for crossed disparity (target patch in front of the adjacent reference patches) and the other for uncrossed disparity (target patch behind the references). The trials were divided into triplets: three correct responses decreased the disparity magnitude by one unit step, two correct responses left the disparity unchanged and only one or zero correct response increased the disparity by two unit steps. Stereo threshold was estimated as the disparity at the 84% correct response rate (*d*′=1), obtained by fitting a Probit function. The threshold reported for each observer is the average threshold estimate from two blocks of measurements (training session: 275 trials/block; pre- and post-training sessions: 160 trials/block).

### 2.6 General experimental design

The experiment generally consisted of three segments: pre-training measures, training and post-training measures. In the pre-training segment, observers were tested with 3 (Experiment 1) or 4 (Experiment 2) different stimulus conditions, including different spatial frequencies (from 1.25 to 20 cpd) and carrier orientations (horizontal and vertical). In the training segment, observers were trained with a specific stimulus condition for 13 sessions. The post-training segment was identical to the pre-training segment. Each training session consisted of a total of 550 response trials (crossed disparity, 250 trials; uncrossed disparity, 250 trials; catch trials or zero disparity, 50 trials) in about 45 min. The experiment was self-paced, and a break was given whenever the subject requested one. The participants were naive to the purposes of the experiments and none of them had any prior experience in psychophysical experiments. It should be noted that our visual task is easy to comprehend, much like the standard stereopsis screening tests used in clinical settings, and participants were given practice trials (roughly 50 trials) to familiarize them with the stimuli and methods prior to the baseline measurements.

## 3. Results

### 3.1 Learning to improve three-dimensional vision

**Figure 2.**
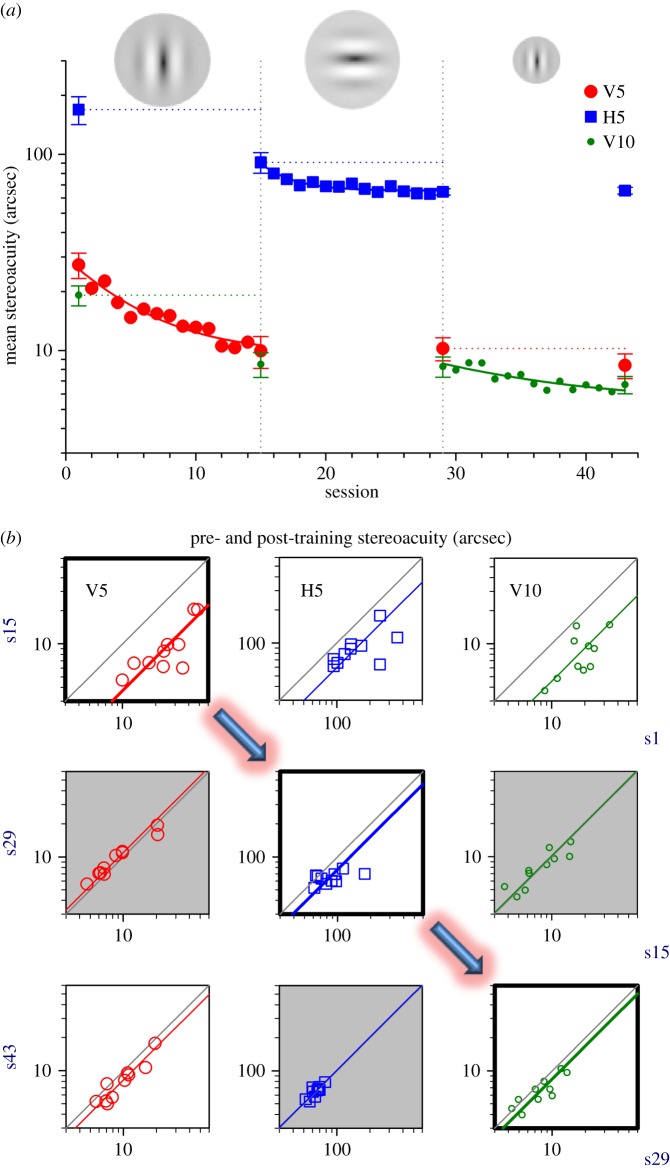
Experiment 1. Perceptual learning of stereoacuity: specificity for carrier orientation and spatial frequency. (*a*) The training protocol consisted of three training stages: stage 1, V5 (vertical carrier: 5 cpd); stage 2, H5 (horizontal carrier: 5 cpd); stage 3, V10 (vertical carrier: 10 cpd). A 3-parameter exponential function was used to quantify the learning profile. Mean thresholds (*n*=10) for each of the three stimulus configurations were measured before and after each training stage. Error bars indicate the standard error of the mean unless stated otherwise. (*b*) The pre- and post-training threshold data of individual observers (*n*=10) are illustrated in the nine figure panels: 1st row, s15 versus s1; 2nd row, s29 versus s15; 3rd row, s43 versus s29. Arrows indicate the sequence of direct training from stage 1 to 3. White panel area denotes statistically significant. Grey panel area, not statistically significant. Note that the abscissa label for each row is displayed at the right-hand end of all the panels highlighted in dark blue.

In the first experiment, we asked whether practicing a stereoscopic depth detection task enhances stereoacuity in adults with normal vision and whether the learning effects, if any, transfer across different stimulus configurations. Ten adults with corrected-to-normal vision participated. The training protocol consisted of three stages, each of 13 sessions (figure 2*a*). In stage 1, participants were trained with targets with a vertical carrier of five cycles per degree (V5: vertical, 5 cpd). In stage 2, they continued to train with the same spatial frequency, but with an orthogonal carrier orientation (H5: horizontal, 5 cpd). They were subsequently required to practice with targets with a vertical carrier at a higher spatial frequency (V10: vertical, 10 cpd) in stage 3. Thresholds for each of the three stimulus configurations were measured before and after each training stage. Each training session consisted of about 500 trials.

We found that practice significantly enhances stereovision. On average, our observers showed almost a factor of three improvement (post/pre, 64%) in mean stereoacuity (red circles, from 27.3 (session 1, s1) to 9.9 arcsec (session 15, s15); paired-*t*=6.510, *p*<0.001) after practicing with V5 stimuli in stage 1. A 3-parameter exponential function, *Th*=*Th*_i_×e^(−*bx*)^+*Th*_p_, was used to quantify the learning profile, where *Th* is stereo threshold, *Th*_i_ is the initial threshold, *Th*_p_ is the plateau threshold and *x* is the training session. Asymptotic performance was obtained in roughly ten sessions (approx. 5000 responses). The pre- and post-training data of individual observers are displayed in figure 2*b*. The top left panel illustrates the threshold data for V5 direct training (s1 versus s15). Note that the area below the grey 1 : 1 reference line represents improvement in stereoacuity. For clarity, the error bars for all individual training sessions were omitted.

### 3.2 Specificity to stimulus configurations

We examined the specificity of visual learning; i.e. whether improvement transfers to a different orientation or a different spatial frequency, in order to further explore the possible mechanisms for the plasticity. To test whether the visual learning effects transfer to the untrained stimulus orientation, we compared pre- and post-training measurements of thresholds with horizontal carriers (H5, figure 2*a* inset). To examine the specificity of the visual learning to the trained stimulus spatial frequency, we performed pre- and post-training measurements of thresholds with carriers of higher spatial frequency, by a factor of 2 (V10, figure 2*a* inset).

Learning generalized substantially to the untrained stimuli. Practicing with V5 stimuli resulted in improvements of 46% for H5 (blue squares, from 169.1 to 91.0 arcsec; s1 versus s15: paired-*t*=3.322, *p*=0.009) and 56% for V10 (green circles, from 19.1 to 8.5 arcsec; s1 versus s15: paired-*t*=6.042, *p*<0.001). The raw training data for individuals are shown in figure 2*b* (first row, middle and right panels).

### 3.3 Transfer: complete or incomplete?

To quantify the transfer of learning, we calculated a Transfer Index (*T*=PI_untrained_/PI_trained_) for individual observers, where PI is the per cent improvement, *T*=1 indicates complete transfer and *T*=0 indicates no transfer. Mean *T*=0.67±s.e. 0.11 and 0.88±s.e. 0.09 for the orthogonal orientation (H5) and higher spatial frequency (V10), respectively. These values suggest nearly complete transfer of learning; however, it is not entirely clear whether the performance on the trained stimulus had reached the respective plateau levels or whether additional direct training could further boost the already sharpened stereoacuity induced by indirect learning.

To answer that question, the participants were asked to engage in subsequent direct training with the two previously untrained stimuli. In stage 2, they continued to train with H5 stimuli for another 13 sessions and interestingly, they continued to improve substantially with practice. The mean improvement relative to session 15 was 29% (s15 versus s29: paired-*t*=2.653, *p*=0.026), resulting in a total improvement (from s1) of 63%. Individual data can be found in figure 2*b* (second row, middle panel). However, no significant change in stereoacuity was observed for V5 or V10 stimuli (second row, left and right panels; s15 versus s29: paired-*t*<0.481, *p*>0.642).

In stage 3, the participants were trained with V10 stimuli for 13 sessions. Similar to the results of stage 2, the enhanced performance resulting from indirect learning was further improved following subsequent direct training, with mean acuity improvement of 19% (figure 2*b*, third row, right panel; s29 versus s43: paired-*t*=3.017, *p*=0.015), resulting in a total improvement (from s1) of 64%.

It is worth noting that all participants completed training with V5 stimuli over a large number of sessions and had already given a total of approximately 7000 responses in stage 1, and their performance levels appeared to be very stable at the end of the training course. Surprisingly, practicing V10 stimuli slightly, but significantly boosted the previously ‘plateaued’ V5 performance, with mean improvement of 18% from 10.2 to 8.4 arcsec (figure 2*b*, third row, left panel; s29 versus s43: paired-*t*=3.928, *p*=0.003). No further significant change was found for the orthogonal H5 stimuli (figure 2*b*, third row, middle panel; s29 versus s43: paired-*t*=0.587, *p*=0.572).

In brief, additional significant improvements obtained with subsequent direct training in stages 2 and 3 evidently suggesting that the transfer of learning observed in stage 1 may not have been complete. Those extra improvements were specific to the difference in stimulus characteristics. We speculate that practice with feedback fine-tunes the internal template to better sample, or learn, the visual stimulus [25], allowing more precise processing of stereoscopic depth information.

### 3.4 Bandwidth of learning

Here our findings reveal that stereoacuity learning can transfer across the spatial frequency spectrum, from the trained spatial frequency to the untrained ‘neighbour’ spatial frequency. When trained at a medium spatial frequency (5 cpd), substantial acuity improvement was recorded at one octave higher (10 cpd). However, these two spatial frequencies likely fall within the same spatial frequency selective mechanisms, as these putative channels have a bandwidth of one to two octaves [26–29]. How broad is the bandwidth of transfer?

To quantify the bandwidth of generalization of learning across spatial frequency, another set of 21 adult participants with normal vision were randomly assigned into two groups (LH, *n*=10; HL, *n*=11). In stage 1, group LH was trained at a low spatial frequency (L, 1.25 cpd) and group HL was trained at a high spatial frequency (H, 20 cpd). In stage 2, observers crossed over and trained at the untrained spatial frequency (group LH, 20 cpd; group HL, 1.25 cpd). Thresholds were measured at each of four spatial frequencies (1.25, 5, 10 and 20 cpd) before and after each training stage. Observers practiced for 16 000 trials over 29 sessions. Trial-by-trial feedback to response was provided.

### 3.5 ‘Coarse-to-fine’ spatial frequencies

**Figure 3.**
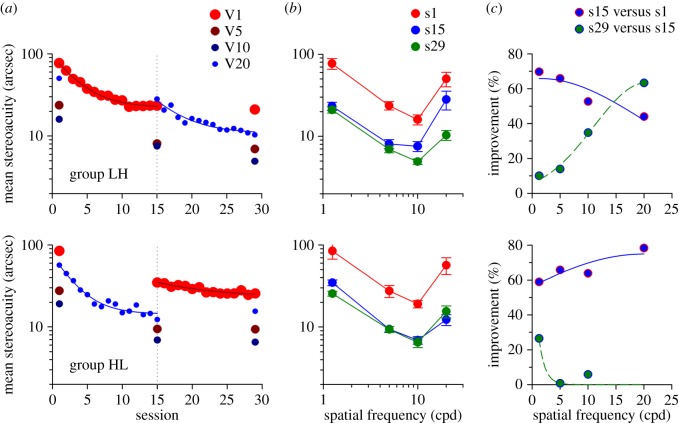
Experiment 2. Bandwidth of generalization across the spatial frequency spectrum. (*a*) Mean stereoacuity as a function of session. In stage 1, group LH (first row, *n*=10) was trained with V1 stimuli and group HL (second row, *n*=11) was trained with V20 stimuli. In stage 2, observers crossed over and trained at the untrained spatial frequency: group LH, V20; group HL, V1. A 3-parameter exponential function was used to quantify the learning profile. (*b*) Mean stereoacuity across spatial frequencies. (*c*) Per cent improvement in mean stereoacuity (*I*) as a function of spatial frequency (*f*). A 3-parameter Gaussian function, *I*=*I*_*t*_×e^−(1/2)(*f*−*f*_*t*_/*σ*)^2^^, was used to quantify the generalization of stereoacuity learning across spatial frequencies, where *I*_*t*_ is the per cent improvement occurring at the trained spatial frequency (*f*_*t*_) and *σ* denotes standard deviation.

We found that the relatively coarse stereopsis evident at low spatial frequencies can be substantially enhanced with repetitive practice. The observers in group LH showed a more than threefold (70%) improvement in mean stereoacuity (red circles, from 77.0 to 23.3 arcsec; s1 versus s15: paired-*t*=5.526, *p*<0.001) after practicing with V1 stimuli in stage 1 (figure 3*a*, top panel); asymptotic performance was achieved in roughly 10 sessions. Interestingly, all three untrained higher spatial frequencies also improved substantially (figure 3*b*, top panel; V5, V10 and V20: paired-*t*>3.712, *p*<0.005). The threshold data are replotted as percentage improvement in figure 3*c*. It is important to note that the transfer was progressively reduced as spatial frequency increased (blue symbols); however, a 44% improvement (a *T* of approx. 0.63) was still obtained for V20 stimuli, 4.3 octaves from the trained spatial frequency and quite close to the visual acuity limit.

We fitted the threshold versus spatial frequency data with a 3-parameter Gaussian function of which the centroid position (mean) was constrained to be at the trained spatial frequency. The bandwidth of transfer, which was defined as the half-width at half height ($B=\sqrt{2\ln2}\cdot \sigma ,$ where *σ* denotes standard deviation) from low frequency (1.25 cpd, approx. Snellen 20/480) to high frequency (20 cpd, approx. Snellen 20/30) was calculated as 23.2 cpd. In other words, the effect of transfer decreased by approximately 40% at 1 s.d. away from the trained spatial frequency. In short, these findings show that practicing with low spatial frequency stimuli enhanced coarse stereopsis and importantly, the learning effects extend broadly towards the high spatial frequency range.

To address the question of whether the transfer was complete or not at high spatial frequencies, the participants continued to train with V20 stimuli in stage 2 (figure 3*a*). A further significant improvement of as much as 63% was observed with subsequent direct training (figure 3*b*, top panel; s15 versus s29: paired-*t*=2.882, *p*=0.018), pointing to an incomplete transfer. Similar to the first experiment, we observed some smaller indirect learning effects for lower spatial frequency stimuli (figure 3*c*, top panel, green symbols)—the improvement was significant for V10 stimuli (s15 versus s29: paired-*t*=2.833, *p*=0.020). Surprisingly, there was also a small improvement for V1 stimuli, 10% beyond the ceiling, although this was not statistically significant (s15 versus s29: paired-*t*=1.610, *p*=0.142). Note the performance appeared to have reached a fairly stable ‘plateau’ level after a large number of training trials in stage 1.

It is worth pointing out that the transfer of learning in stage 2 (figure 3*c*, blue curve, with the peak at the trained frequency) was in the opposite direction from what we found in stage 1 (green curve) in which the transfer was induced from low to high spatial frequency. Those participants were first trained with V1, leaving little room for improvement, and that is probably why the bandwidth of transfer obtained in stage 2 (11 cpd) was much narrower than that obtained in stage 1 (23.2 cpd).

### 3.6 ‘Fine-to-coarse’ spatial frequencies: asymmetric transfer

In order to quantify the characteristics of transfer from high to low spatial frequency, another group of participants (group HL) was first trained with V20 stimuli in stage 1. Direct training resulted in a more than threefold (78%) improvement in mean stereoacuity (figure 3*a*, bottom panel; s1 versus s15: paired-*t*=3.727, *p*=0.004) and the learning effect transferred to all three untrained lower spatial frequencies as well (figure 3*b*, bottom panel; s1 versus s15: paired-*t*>2.804, *p*<0.019).

Interestingly, we observed an asymmetric transfer of learning across the spatial frequency spectrum. The bandwidth of transfer induced by training with V20 stimuli appeared to be broader than that induced with V1 stimuli (figure 3*c*, top panel versus bottom panel, blue curve; approximately 35%, 31.1 versus 23.2 cpd), meaning that larger improvements were obtained for the other three untrained spatial frequencies. There was a 59% acuity improvement four octaves away from the trained spatial frequency. By contrast, at the same octave distance the improvement was smaller (44%) for group LH. An additional improvement of 26% was obtained with subsequent direct training with V1 stimuli (s15 versus s29: paired-*t*=4.092, *p*=0.002), with no further significant change observed for the other three higher spatial frequencies. Note that the effect of direct training observed here was substantially weaker when compared with group LH in stage 2 (figure 3*c*, top panel versus bottom panel, grey dashed curve; HL_V 1,s29_/HL_V 1,s15_ versus LH_V 20,s29_/LH_V 20,s15_: unpaired-*t*=3.267, *p*=0.004), as a consequence of a stronger transfer effect in stage 1. All these findings clearly indicate that the transfer of learning was stronger when going from high to low spatial frequency than going in the opposite direction.

## 4. Discussion

**Figure 4.**
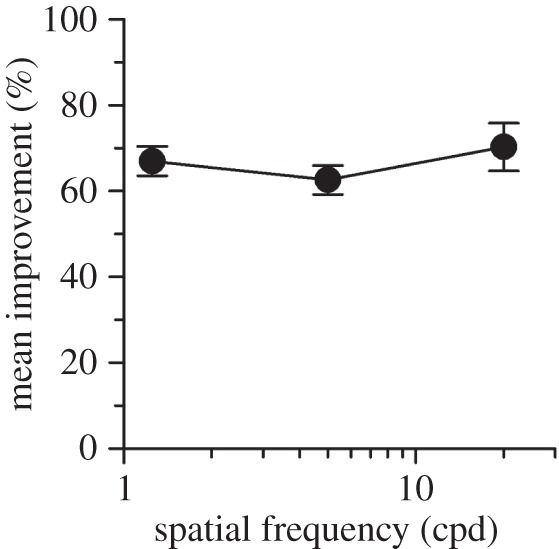
Enhancing coarse-to-fine stereoacuity with perceptual learning. Mean per cent improvement in stereoacuity resulting from direct training as a function of spatial frequency (stage 1 of the two experiments; V5 from the first experiment; V1 and V20 from the second experiment). There was no significant difference in per cent improvement among the three frequency groups (ANOVA: *F*=0.795, *p*=0.461).

We investigated the spatial frequency selectively of perceptual learning of stereopsis, finding approximately 60–70% improvement in stereoacuity across a broad range of frequencies, from as low as 1 cycle per degree to near the resolution acuity limit (figure 4). The spatial frequency bandwidth of stereo perceptual learning (≈4 octaves) is very much broader than the ≈1.4 octave bandwidth of contrast sensitivity learning in normal observers [30].

Surprisingly, the improvement also generalized to the orthogonal orientation. With Gabor stimuli, such as those used here, stereoacuity with vertical carrier gratings is highly precise, since the vertical contours provide the necessary horizontal disparity information for making fine stereoscopic judgements. By contrast, with horizontal carrier gratings stereoacuity is coarse—almost a log unit worse than with vertical carriers—because the observer must use the Gaussian envelope to make the depth judgement. Thus, it is remarkable that the learning generalizes across orientations.

This broad transfer of depth discrimination learning contrasts with the highly specific learning that is found in other basic visual functions such as discrimination of contrast, orientation, spatial frequency, direction, depth, Vernier and texture segmentation [31]. The broad transfer reported here is also at odds with the highly orientation-specific learning [15] that has been reported with random dot stereograms. We have no ready explanation for this discrepancy; however, methodological issues are important in perceptual learning [32]. Random-dot stereograms may represent a much more difficult task compared with our contour stereoacuity test, because many observers have difficulties seeing these type of stereograms [19]. As task difficulty increases, learning can become more specific to stimulus features [13]. However, one possible explanation is that our contour stereoacuity test provides two sources of depth information (i.e. envelope and carrier cues), whereas random-dot stereograms provide only a single source of depth information.

Broad transfer occurred even when the visual stimuli differed in appearance from the previously trained ones (for example, see the three stimulus conditions displayed in figure 2). However, the nature of the task remained essentially the same: to determine the stereoscopic depth of the target, relative to the surround. This broad generalization suggests that the learning occurred in the higher level cortical areas, beyond the point of binocular convergence [33].

A close look at the learning curves in figures 2 and 3 reveals that the session-by-session improvements were smooth and gradual, requiring more than a total of 5000 response trials to reach a plateau, suggesting that this was genuine perceptual learning rather than the result of instrumental or cognitive learning (i.e. learning to do the task). Similar learning profiles have been reported for learning contour [6] and random dot stereograms [34,35], with considerable inter-observer variability of learning [36]. Although we cannot completely rule out the possibility that in part the improved stereoacuity might reflect improved vergence, and thus reduced vergence noise, previous work has demonstrated that stereopsis is robust to disjunctive eye movements [37]. We believe that the enhanced visual performance reflects the effects of genuine neural plasticity triggered by perceptual learning.

The issue of specificity/generalization has attracted wide attention in the field of perceptual learning [38], both for theoretical and practical reasons. Perceptual learning would not be practical for clinical use [39–42] if the learning effects were very specific to the trained stimulus features. In that case, it would be necessary to perform training with a large number of visual targets of different sizes, spatial frequencies, orientations, shapes, etc., in order to reap the benefits of improved stereopsis in the real world.

How can we optimize perceptual learning in order to maximize visual performance? Our findings provide some hints. One surprising finding is that the apparently stable ‘learning plateau’ for lower frequency stimuli (V5 (figure 2, stage 1) and V1 (figure 3, stage 1)) can be further stretched by subsequent training with higher frequency stimuli (V10 (figure 2, stage 3) and V20 (figure 3, stage 2), respectively). One simple explanation is that the observers had not yet reached a performance plateau in stage 1, but we believe that was not the case. First, in a preliminary study adopting an extended training protocol consisting of 25–30 sessions, we found that 10–12 sessions were sufficient to reach a performance plateau for stereoacuity; therefore, we decided to do 15 sessions in the present experiments. Second, regression analysis (V5: 16 sessions, s1–15 and s29) with an additional plateau parameter (*r*^2^=0.925) provides a better fit than a 2-parameter model with no plateau parameter (*r*^2^=0.891), further supporting the occurrence of the performance plateau during the course of learning. Third, we note that it is not simply the extra number of sessions that matter. When the orientation of the carrier gratings was orthogonal in the subsequent training (H5 (figure 2, stage 2)), no further enhancement beyond the ceiling was obtained for V5.

This ‘beyond-the-plateau’ learning appears to be specific to stimulus orientation. As shown in figure 2, the subsequent training with finer stimuli (i.e. V10) actually transferred back only to those previously learned coarser gratings with the same carrier orientation (i.e. V5), but not to the orthogonally oriented ones (i.e. H5). Similar orientation-specific ‘relearning’ was observed for horizontally oriented stimuli (i.e. H5) following generalization from practicing with vertically oriented stimuli (i.e. V5). We also found that despite substantial transfer from low to high spatial frequencies (i.e. figure 2*a*, V5; figure 3*a* top panel, V1), subsequent direct training resulted in a small further improvement in stereoacuity for higher spatial frequencies.

Thus, while there is broad transfer of stereo learning in the first phase, it appears that supplementary direct training may be necessary when optimizing perceptual learning across orientations. Interestingly in most cases, the time needed to achieve plateau performance was quicker than the initial learning (figures 2*a* and 3*a*, stage 1). Similarly, earlier studies have reported that considerable practice is necessary when learning new stereo stimuli with different shapes [17,19,20].

We found asymmetric transfer of stereo learning. The bandwidth of transfer was broader when training was at a high spatial frequency than at a low spatial frequency (figure 3*b*). Task difficulty [13,43] and task precision [44] can strongly influence the generalization of learning. Perhaps the higher frequency task with lower thresholds represents an easier task compared with the lower frequency one, thereby facilitating the transfer.

The specificity of perceptual learning for low-level stimulus features has often been taken as reflecting neural alterations at the early stages of cortical processing [33]. Alternatively, with more knowledge about the stimulus configuration acquired during practice, observers can construct a more efficient, better-matched template [25] at the higher cortical levels to more accurately localize the visual target presented to each eye and compute binocular disparity. Previous studies have shown that selective spatial attention mechanisms can explain the learning generalization to untrained retinal locations [18,21]. Notably, a double training technique has been demonstrated to be useful in enabling transfer of learning across retinal locations or orientations through top-down processes [45,46]. In agreement with our results, recent research suggests that perceptual learning can arise from different levels of visual processing: decision rules, attentional learning and physiological changes [32]. Changes in training procedures can cause a preference shift from one of those mechanisms to another.

## 5. Conclusion

In short, both coarse and fine stereopsis can be enhanced by perceptual learning. Using narrow-band luminance spatial frequency stimuli, we were able to systematically characterize the time-course, magnitude and specificity of stereoacuity learning over a large range of spatial scales, and revealed useful strategies to boost the learning outcomes. The multi-stage training protocol allowed different levels of visual processing to be isolated, providing new insights into the learning mechanisms of stereopsis. For practical purposes, one of the most important findings is the asymmetric transfer of learning across the spatial frequency spectrum. Learning transfers more efficiently from high to low spatial frequencies than from low to high. The ability to generalize efficiently may provide a key principle for triggering neural plasticity and restoring impaired binocular vision in clinical situations.

## Data accessibility

Data files relevant to this study can be found in the Dryad database (http://dx.doi.org/10.5061/dryad.3td7f).
